# Comparison of methods and characterization of small RNAs from plasma extracellular vesicles of HIV/HCV coinfected patients

**DOI:** 10.1038/s41598-020-67935-1

**Published:** 2020-07-07

**Authors:** Elena Martínez-González, Óscar Brochado-Kith, Alicia Gómez-Sanz, Luz Martín-Carbonero, Ma Ángeles Jimenez-Sousa, Paula Martínez-Román, Salvador Resino, Verónica Briz, Amanda Fernández-Rodríguez

**Affiliations:** 1 Unit of Viral Infection and Immunity, National Center for Microbiology, Institute of Health Carlos III, Carretera Majadahonda- Pozuelo, Km 2.2, Majadahonda, 28220 Madrid, Spain; 2 Hospital La Paz Institute for Health Research (IdiPAZ), Madrid, Spain

**Keywords:** Hepatitis C virus, Viral immune evasion, Cellular microbiology, HIV infections, Transcriptomics

## Abstract

Hepatitis C virus (HCV) and human immunodeficiency virus (HIV) hijack the host exosomal machinery as an additional mechanism of infection and evasion of the immune system, modifying the small RNA (smRNA) cargo during infection. We characterized the surface epitopes of extracellular vesicles (EVs) from plasma HIV/HCV-coinfected patients and their smRNA cargo profile, by comparing different isolation procedures. Six EVs isolation procedures were compared: ultracentrifugation, and five different polyethylene glycol-based methods (commercial, combined with a column purification step and two custom); and two RNA commercial kits (phenol and non-phenol based) were used. High-throughput sequencing of smRNAs was performed. Exosomal surface epitopes were analyzed by the MACSPlex Exosome Kit. Four miRNAs displayed differences among protocols (hsa-miR-205-5p and hsa-let-7a/b/f-5p). The selection of RNA isolation kit impacted on the detection of miRNAs and other smRNAs, where the phenol-based RNA isolation kit performed acceptably. EVs surface was enriched with HLA-DR/DP/DQ, CD81, and CD8. There were three liver-specific miRNAs overexpressed (let-7a-5p, miR-21-5p and hsa-miR-122-5p), thus, EVs cargo might reflect liver disease evolution. Other smRNAs such as piwi-interacting RNAs were also detected for the first time. Custom polyethylene glycol precipitation-based methods combined with an RNA phenol-based kit yielded the higher number of smRNAs for EVs isolated from plasma HIV/HCV patients.

## Introduction

Around 36.7 million people are globally living with human immunodeficiency virus (HIV) infection. It is estimated that approximately 2.3 million of these patients are also coinfected with hepatitis C virus (HCV)^1^. The risk of coinfection with HCV among HIV-infected patients is elevated because both viruses share routes of transmissions and behavioral patterns. Liver disease has become the leading cause of morbidity and mortality within HIV/HCV coinfected patients^1^. HIV/HCV-coinfected patients are prone to have advanced liver disease and more accelerated progression than HCV-monoinfected individuals. Moreover, the presence of HIV modifies the natural cycle of HCV infection, making higher HCV titers, progressing faster to cirrhosis, liver fibrosis, and hepatocellular carcinoma, among others^2^.

Extracellular vesicles (EV) like exosomes are involved in both HCV and HIV infections. Exosomes are small (~ 100 nm) vesicles containing proteins, mRNAs, and smallRNAs (smRNAs) as cargo. They are released into the extracellular milieu by practically all eukaryotic cell types are found in most biological fluids. Different cell types can take up exosomes by endocytosis, and thus their microenvironment can be modified. EVs play, therefore, an essential role in cell-to-cell communication, affecting both physiological and pathological processes, including liver diseases^3,4^, and showing great potential for development as diagnostic or therapeutic tools. On the other hand, virally-infected cells can secrete different types of RNAs in exosomes to cause latent infection and propagation to nearby cells. Thus, exosomes can accelerate virus infection as it evades the neutralization by antibodies allowing a better virus spread^5^*.* The released exosomes carry non-coding RNAs (ncRNAs) such as smRNAs, including microRNAs (miRNAs), piwi-interacting RNAs (piRNAs), small nucleolar RNAs (snoRNAs), small nuclear RNAs (snRNAs), ribosomal RNAs (rRNAs), and long non-coding RNAs, as well as DNA and cytokines, among others. The smRNAs are being examined as potential biomarkers for various diseases for their high stability within vesicles, as they can affect gene expression in the recipient cells^6^, especially miRNAs.

As HIV and HCV hijack the machinery of exosome biogenesis to promote viral infection, exosome profile can be altered in HIV/HCV-coinfected patients^7^. HCV may infect their target cells in two ways: cell-free viruses and through cell-to-cell contact (exosomes). In fact, HCV RNA has been detected in exosomes, which could elicit the innate immune response in dendritic cells^4,8^. This last cell-to-cell mechanism is also used by HIV to evade immune surveillance, resulting in an accelerated infection and dissemination^9^. Also, HIV modifies exosomal cargo, deregulating miRNAs and proteins in exosomes released by infected cells. Thus, as the miRNA expression reflects the physiological or pathological state of the original cell, the study of the exosome miRNA profile may give us insight into the altered molecular pathways and disease condition in HIV/HCV coinfection^4,7^. Therefore, the study of EVs can help finding useful biomarkers candidates for the identification or diagnosis of liver disorders. Exosomal biomarkers yield high sensitivity, specificity, and also excellent stability. In this context, EVs are emerging as potential non-invasive diagnostic and therapeutic tools in infectious diseases^10^; and their cargos can be associated with liver disease status and treatment responses, among others^7^.

Nevertheless, some challenges in the study of these vesicles remain since isolation and quantitative analysis of EVs requires sophisticated and more evolved technologies. Nowadays, there is a big offer of different protocols to isolate plasma exosomes, but does not exist a suitable standard procedure for each study, or widely accepted. Traditionally, differential ultracentrifugation (UCF) has been widely used for isolating exosomes from biological fluids, but a number of commercial kits have been launched to isolate and study exosomes for various purposes, which are usually less time consuming, less technique sensitive, more compatible with limited volumes and less special equipment requirements^11^. In addition, polyethylene glycol (PEG) precipitation-based protocols, which have been traditionally used for virus isolation, have also been applied to purification of exosomes. This is an inexpensive alternative to commercial kits, which is less known. Each procedure will affect downstream applications, being essential to a correct selection of the method based on further analysis. Moreover, the selection of the method for RNA analysis inevitably affects the quantity and type of RNA isolated. Some experts have suggested a combination of several separation techniques, but no consensus has been reached yet. However, the use of different separation techniques supposes higher cost, separation steps, and time-consuming; which is not affordable for clinical research^12^, where clinical samples are limited and large number of samples need to be processed in a uniform and efficient manner.

Facing the lack of plasma EVs information of HIV/HCV patients, we performed for the first time a characterization of the surface epitopes and the smRNA cargo of HIV/HCV coinfected individuals. We also compared twelve different experimental strategies for their application to clinical practice, analyzing the performance of each protocol to perform smRNA profiling in HIV/HCV patients.

## Results

**Figure 1:**
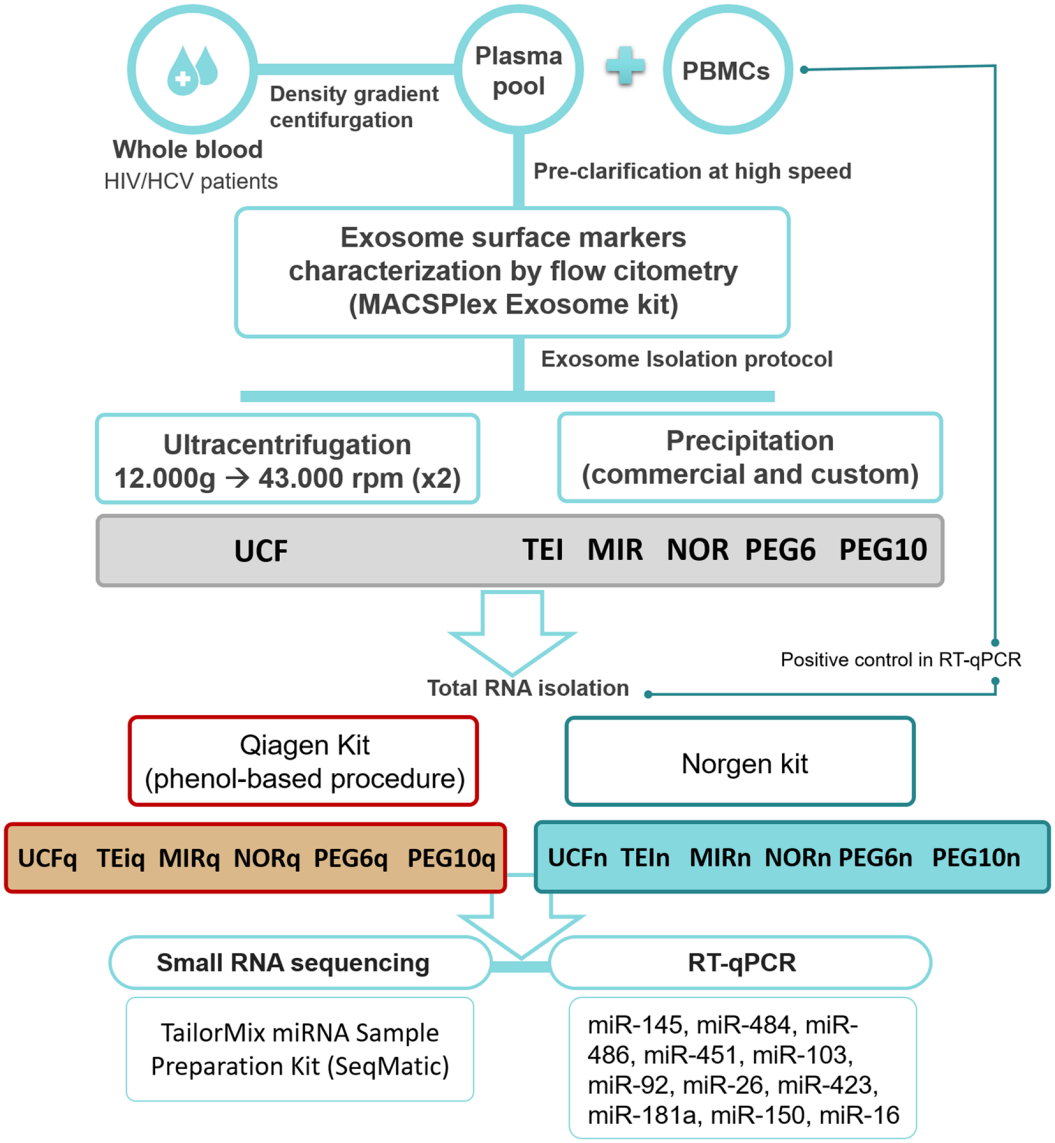
Flow chart of the experimental procedure.

Epidemiological and clinical characteristics of the four patients are shown in Table 1. All patients were Caucasian, HIV/HCV coinfected, aviremics for HIV, none of them were previously treated for HCV, 50% were male, the median age was 52.5 years, and non-liver fibrosis (F0–F1) was reported. Three patients were infected by HCV genotype 1 and one by genotype 4. A flow chart of the experimental procedure is shown in Fig. 1.

**Table 1:** Epidemiological and clinical characteristics of each patient pooled.

Patients characteristics	Patient 1	Patient 2	Patient 3	Patient 4
**Gender**	Female	Male	Female	Male
**Age (years)**	52	47	54	53
**Ethnic group**	Caucasian	Caucasian	Caucasian	Caucasian
**HIV infection**	HIV-1	HIV-1	HIV-1	HIV-1
Route of transmission	IDUs	Sexual	IDUs	IDUs
Viral subtype	Unknown	B	Unknown	Unknown
HIV viral load (Ul/ml)	Undetectable	< 20	< 40	Undetectable
Clinical stage	B3	A1	B3	B3
Antiretroviral therapy	ABC/DTG/3TC	ABC/DTG/3TC	3TC + DRV/c	ETR + DRV/r
Viral tropism	No	No	No	No
CD4 (%)	26	36	30	29
**Chronic HCV infection**	Yes	Yes	Yes	Yes
HCV route of transmission	IDUs	Sexual	IDUs	IDUs
Genotype and viral subtype	1a	1a	1	4
HCV active infection	Yes	Yes	Yes	Yes
HCV viral load (Ul/ml)	786,000	353,000	74,300	10,000,000
Fibrosis stage	F0–F1	Unknown	F0–F1	F0–F1
Fibroscan	6	–	4,3	5,2
**Coinfected with cytomegalovirus**	Yes	Yes	No	Yes
**Hepatitis B**	Resolved	Resolved	Resolved	Vaccinated

*HIV* human immune deficiency virus, *HCV* hepatitis C virus, *IDUs* intravenous drug users, *ABC* abacavir, *DTG* dolutegravir, *3TC* lamivudine, *DRV/c* darunavir/cobicistat, *DRV/r* darunavir/ritonavir, *ETR* etravirine.

### Comparison of exosome isolation procedures for miRNAs and other smRNAs recovery

The raw sequencing data have been included in the ArrayExpress repository (EMBL-EBI) under the accession number E-MTAB-8246.

#### miRNAs

From 2,822 miRNAs recorded in miRBase (v.20), a total of 371 were detected. After filtering and deleting duplicates, 200 miRNAs were selected for subsequent analysis, and 45 miRNAs displayed at least one count per million in all procedures. In terms of the number of identified miRNAs, protocols based on PEG and the miRNeasy kit (Qiagen) showed the highest performance. Thus, protocols based on polyethylene glycol of 10KDa (PEG10), polyethylene glycol of 6KDa (PEG6), and Total Exosome Isolation kit (Thermo Fisher Scientific) (TEI) combined with miRNeasy kit showed a higher variety of miRNAs. Mircury exosome isolation kit—Serum and Plasma (Exiqon) (MIR) and Norgen´s Plasma/Exosome Serum and Free-Circulating RNA isolation Mini Kit (Norgen, Biotek corp.) (NOR), and the RNA isolated with Norgen kit generated, provided on average, the higher number of counts (Supplementary Fig. S1, Supplementary Table S1 and S2).

**Figure 2:**
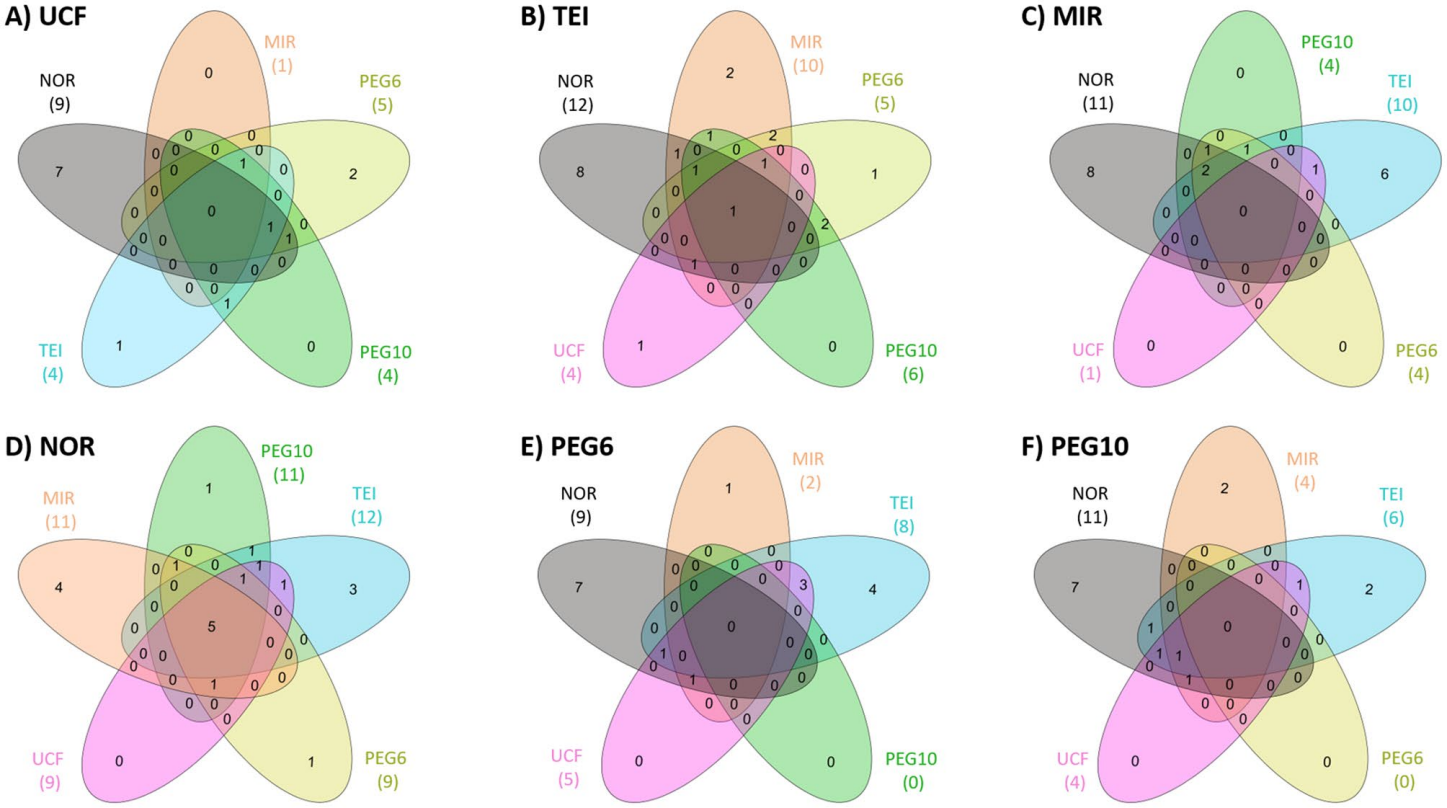
Star Venn diagrams show the overlap of the miRNAs differentially identified between protocols. Each diagram have five ovals which contain the number of miRNAs differentially identified among each protocol versus (**A**) UCF, (**B**) TEI, (**C**) MIR, (**D**) NOR, (**E**) PEG6 and (**F**) PEG10. Overlapping numbers correspond to common miRNAs between different comparisons. Ovals colours correspond to comparison with UCF (pink), TEI (blue), MIR (orange), NOR (grey), PEG6 (yellow) and PEG10 (green).

Statistical analysis showed slight differences between the six exosome isolation protocols, displaying four miRNAs significantly different between methods (Fold change (FC) ≥ 2; False discovery rate (FDR) ≤ 0.05): hsa-miR-205-5p, hsa-let-7a-5p, hsa-let-7b-5p and hsa-let-7f-5p. The protocols with the highest differences with respect to the others were NOR and TEI. We applied a relaxed filtering criterion (FC ≥ 1.5 and *p*-value ≤ 0.05) to explore which miRNAs displayed the bigger differences between protocols without an FDR correction, and these differences were represented for each protocol respect to the others in a Venn diagram (Fig. 2A–F). Thus, we could identify that NOR protocol was the most different to the others, while the custom protocols, PEG6 vs. PEG10, did not display any dissimilarities.

**Figure 3:**
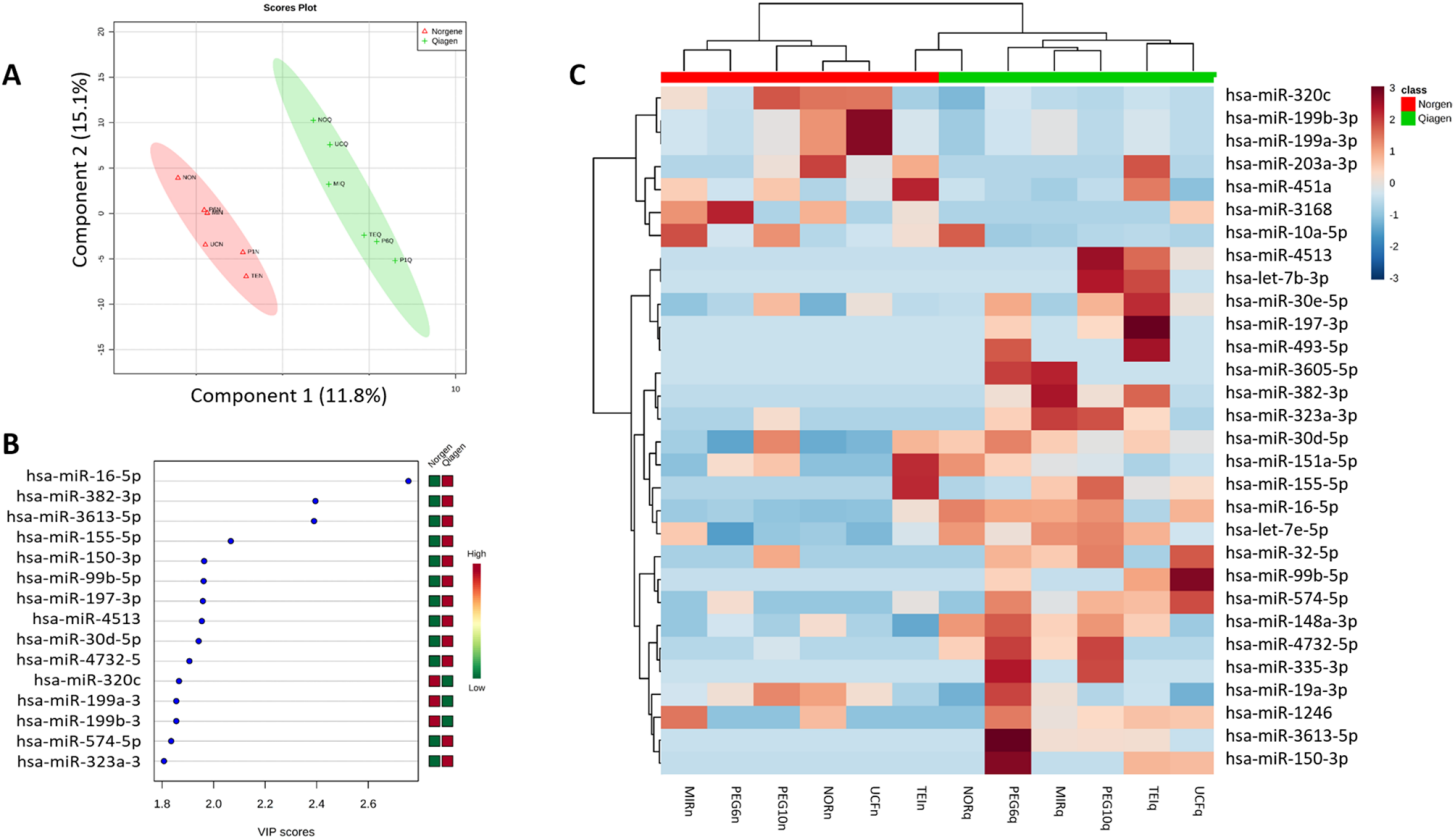
Analysis of miRNAs by RNA isolation kit. (**A**) Multivariate analysis: PLS-DA of miRNAs according to the RNA isolation kit used. Red triangles and green crosses represent samples isolated with Norgene and miRNeasy (Qiagen), respectively. Class membership was represented by a 95% confidence ellipses calculated from PLS-DA scores. (**B**) Variable Importance in Projection (VIP) of the top 15 miRNAs, which represents the relative contribution of miRNAs to the variance between the two RNA isolation kits. The higher the VIP score, the better the contribution of the miRNA to the group separation. The green and red boxes on the right indicate whether the miRNA expression is increased (red) or decreased (green) on each group. (**C**) Heatmap of the 30 top miRNAs in the PLS-DA. Columns represent each sample, while rows correspond to miRNAs. The miRNA clustering tree is shown on the left, and the sample clustering tree is shown at the top. A higher presence of miRNAs is represented by red squares and lower presence by blue squares.

When we analyzed differences between the two RNA isolation kits (miRNeasy of Qiagen vs. Norgen), only the hsa-let-7b-5p showed significant statistical differences (FC > 2 and FDR < 0.05) (Supplementary Table S3). The partial least squares discriminant analysis (PLS-DA) shows us that samples clustered according to the RNA isolation kit used (Fig. 3A), but the predictive ability of classification according to RNA isolation kit used was not significant (R2 > 0.99, Q2 = − 0.41). This analysis shows us that the miRNAs hsa-miR-16-5p, hsa-miR-382-3p, and hsa-miR-3613-5p displayed the highest variable importance in projection (VIP) values (Fig. 3B). The 30 miRNAs with the highest VIP score in the PLS-DA show us that there is a clear different pattern between kits irrespective of the exosome isolation protocol used (Fig. 3C). In general, the miRNAs that most differentiate between RNA isolation kits, showed higher counts with miRNeasy kit (Qiagen).

#### Other smRNAs

Regarding other smRNAs, 1,138 were identified after filtering and were selected for subsequent analysis (56.2% piRNAs, 12.5% rRNAs, 18.4% snRNAs, 12.9% snoRNAs). A total of 67 smRNAs displayed at least one count in all experimental procedures (39 piRNAs, 16 snRNAs and 12 snoRNAs). None of them showed significant differences between exosome isolation protocols with FC > 2 and FDR < 0.05. Next, we applied a relaxed filtering criterium (FC ≥ 1.5 and *p*-value ≤ 0.05) to explore small differences between protocols, and these differences were represented for each protocol respect to the others in a Venn diagram (Supplementary Fig. S2).

**Figure 4:**
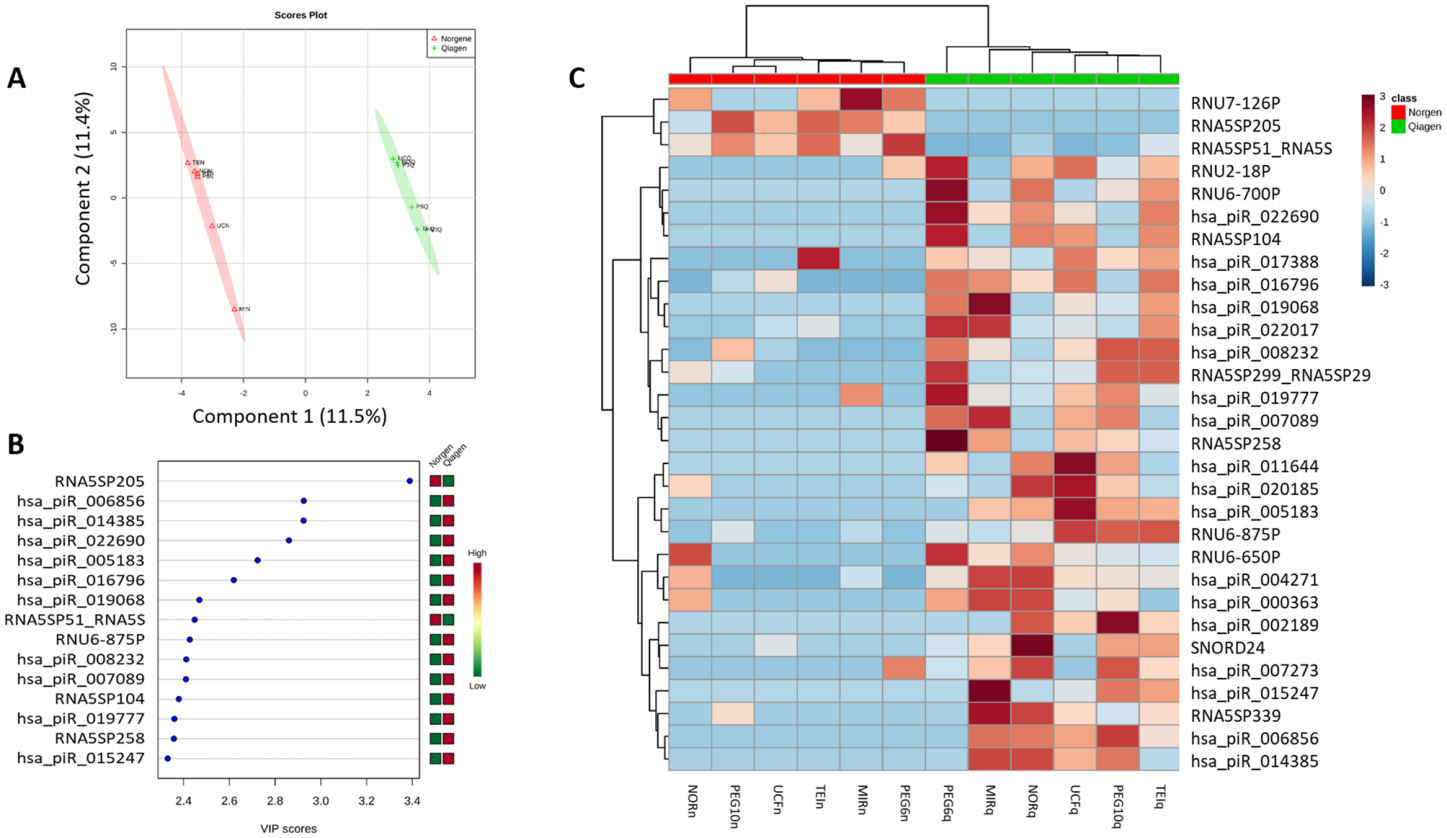
Analysis of smRNAs analysis by RNA isolation kit. (**A**) Multivariate analysis: PLS-DA of smRNAs according to the RNA isolation kit used. Red triangles and green crosses represent samples isolated with Norgene and miRNeasy (Qiagen), respectively. Class membership was represented by a 95% confidence ellipses calculated from PLS-DA scores. (**B**) Variable Importance in Projection (VIP) of the top 15 smRNAs, which represents the relative contribution of smRNAs to the variance between the two RNA isolation kits. The higher the VIP score, the better contribution of the smRNAs to the group separation. The green and red boxes on the right indicate whether the smRNAs expression is increased (red) or decreased (green) on each group. (**C**) Heatmap of the 30 top smRNAs in the PLS-DA. Columns represent each sample, while rows correspond to smRNAs. The smRNA clustering tree is shown on the left, and the sample clustering tree is shown at the top. A higher presence of smRNAs is represented by red squares, and lower presence by blue squares.

Concerning the RNA isolation kit analysis, 40 smRNA were differentially significant between Norgen and Qiagen (Supplementary Table S3). When we analyzed the total normalized counts and the number of identified smRNAs (Supplementary Table S2, Supplementary Fig. S1), the Norgen kit displayed a higher number of counts and miRNeasy the higher number of identified molecules. Figure 4A shows the PLS-DA of smRNA for RNA isolation kit, where samples clustered in two different groups (R2 = 0.99, Q2 = 0.19, three components). The *RNA 5S ribosomal pseudogene 205* (*RNA5sp205*) showed the higher score for discriminating between RNA isolation kits (VIP = 3.4) (Fig. 4B). Finally, the top 30 smRNAs with the highest VIP in the PLS-DA were represented in a heatmap (Fig. 4C), which shows us two main clusters regarding the RNA isolation kit.

#### Proteins

The lowest concentration of total proteins from the exosome lysate samples was obtained with the UCF method (C = 0.17 μg/μl), followed by TEI (C = 0.93 μg/μl) and NOR (C = 1.83 μg/μl). On the other hand, the MIR (C = 14.01 μg/μl), PEG6 (C = 11.73 μg/μl) and PEG10 (C = 6.90 μg/μl) methods showed the higher concentrations.

### Real-time quantitative PCR

We selected eight miRNAs for validation, all of them showed at least one count in all samples (hsa-miR-451a, hsa-miR-103a-3p, hsa-miR-92a-3p, hsa-miR-484, hsa-miR-16-5p, hsa-miR-181a-5p and hsa-miR-423-3p) and the hsa-miR-150-5p, which is usually present in exosomes. The 12 different methods showed different performances in terms of Cq values (Supplementary Fig. S3). For most of the miRNAs, the lowest Cp was obtained with the exosome isolation protocols PEG6 and PEG10 combined with the RNA extraction by Qiagen. The highest Cp values were obtained with the exosome protocol NOR and the RNA isolation kit of Norgen. Therefore, the detection of specific miRNAs by RT-qPCR is influenced by the exosome protocol and RNA isolation kit used.

### Characterization of plasma vesicles from HIV/HCV-coinfected patients

#### Extracellular vesicles surface markers

**Figure 5:**
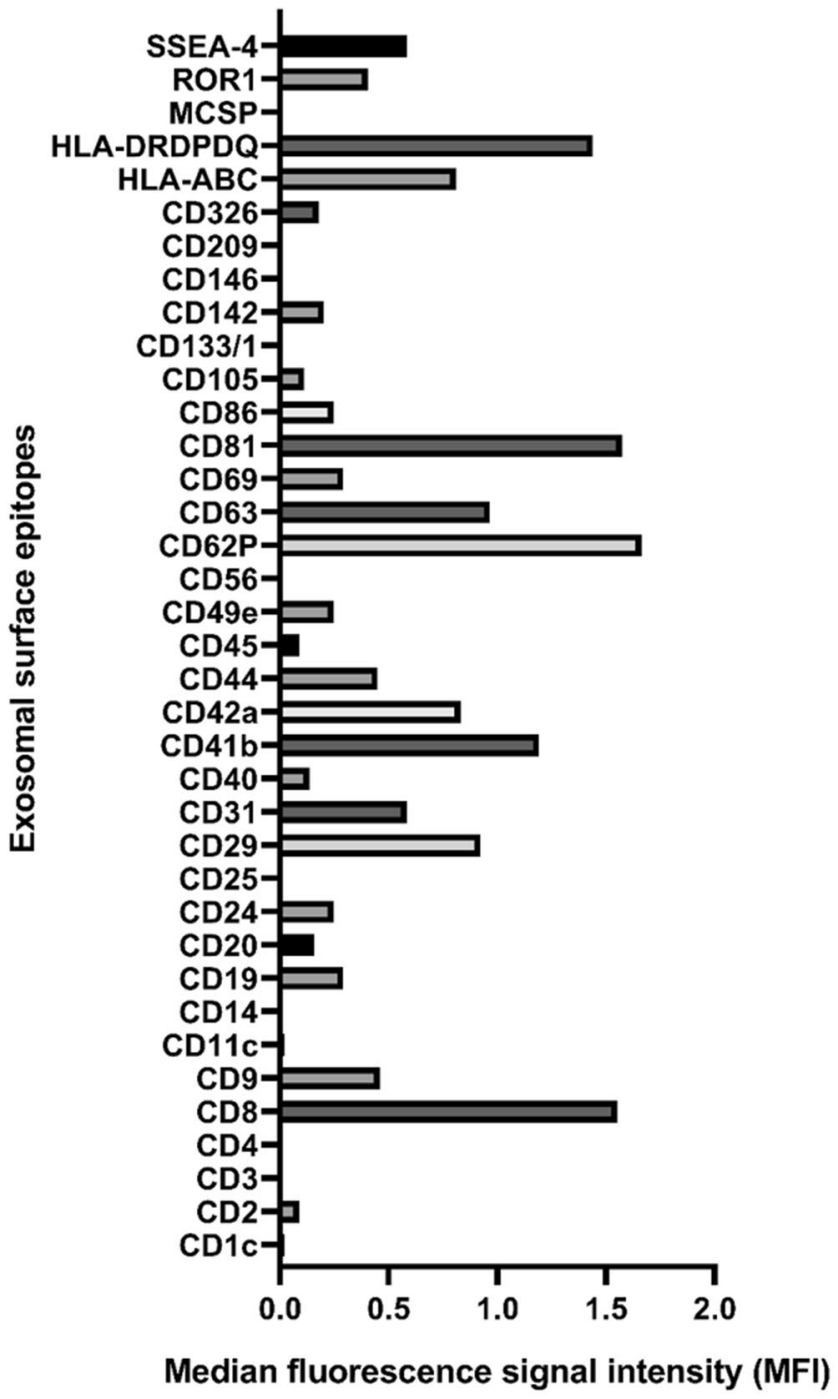
Surface markers of exosomes from a pooled plasma of HIV/HCV coinfected patients. Each bar corresponds to each exosomal surface epitope measured. X-axis indicates the median fluorescence signal intensity (MFI).

We have analyzed the surface markers of exosomes from a plasma pool of HIV/HCV coinfected patients. The analysis with MACSPlex is shown in Fig. 5. The markers that showed higher presence were HLA-DRDPDQ, CD81, and CD8. We also identified a high detection of CD62P (P-selectin).

#### miRNA-related pathways

**Figure 6:**
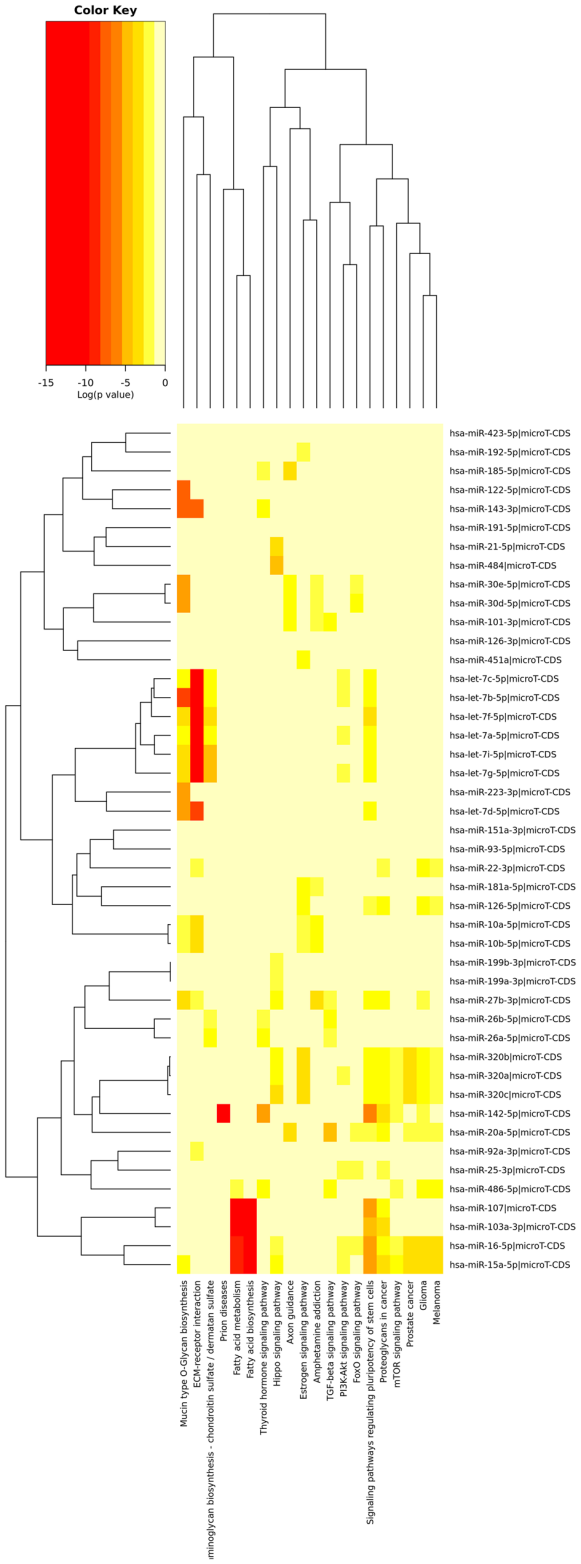
Heatmap viewer of the KEGG pathways union analysis with Tarbase. Over-represented pathways of miRNAs presented in EV of plasma HIV/HCV patients.

After normalization, a total of 45 miRNAs showed at least one count in all experimental procedures. We explored the putative regulated pathways by these miRNAs and identified that mainly fatty acid-related pathways (Fig. 6) were represented. The key miRNAs related to this pathway were hsa-miR-103a-3p, hsa-miR-107, hsa-miR-15a-5p and hsa-miR-16-5p, which regulate *fatty acid synthase* (*FASN*), *acyl-CoA synthetase long chain family member 3* (*ACSL3*) and *4* (*ACSL4*) and the *3-oxoacyl-ACP synthase* (*OXSM*).

The remaining pathways were mainly related to different signaling processes, where the extracellular matrix (ECM) receptor interaction pathway was well-represented by the hsa-let-7 family (a, b, c, f, g and i).

Additionally, liver-specific miRNAs were identified, such as the hsa-miR-let-7a-5p, hsa-miR-21-5p, hsa-miR-22-3p, hsa-miR-199a-3p and the well-known hsa-miR-122-5p.

## Discusion

This study aimed to characterize for the first time the surface markers and the smRNA cargo profile of plasma EVs of HIV/HCV coinfected patients. Twelve different procedures were compared to determine an optimal and transferable technique to clinical practice for biomarkers identification in these patients. As the different experimental procedures presented dissimilar performance, the method used for isolation of EVs should be carefully considered as it can largely influence the final result.

First of all, we have to account for the biophysical and molecular properties of EVs, which are sharing by HIV and HCV particles. This issue makes the precise separation between viruses and exosomes technically challenging. However, as our objective is the smRNA profiling, this is not a drawback. Regarding the smRNA cargo, miRNAs were highly abundant, followed by piRNAs, snoRNAs, snRNAs, and rRNAs. We observed that those based on custom (PEG6, PEG10) and commercial (TEI) polyethylene-glycol precipitation methods, combined with a phenol-based RNA isolation kit (miRNeasy, Qiagen), showed the highest number of different miRNAs and other smRNAs, while the non-phenol based kit of Norgen yielded the highest number of counts. Custom PEG protocols have been used for over fifty years to concentrate and purify viruses, which share some biophysical properties with EVs. Thus, similar procedures have been used to enrich and purify exosomes in an inexpensive and efficient alternative to commercial kits^13^. TEI contains a volume-excluding polymer, which additionally includes a proteinase K treatment to deal with plasma samples when the end goal is the analysis of exosomal RNA or protein cargo. Previous studies in serum samples have identified the best performance of PEG protocols for miRNAs identification, compared with other commercial kits. However, precipitated material may have protein aggregates, which suggests that precipitation methods such as PEG precipitate plenty of non-vesicular components that could interfere in EVs count. However, this is not a drawback if the goal is smRNAs profiling^14^. In terms of counts, the commercial precipitation-based methods MIR and NOR yielded the highest expression for specific smRNAs, although they showed the lower diversity of smRNAs. Moreover, MIR includes a precipitation step with a thrombin treatment to enrich for EVs. Thrombin is a serine protease that converts fibrinogen to fibrin, theoretically making easier an efficient recovery of EVs. Thus, MIR has been recommended as an alternative to UCF for EVs analysis in serum samples^11^. However, others studies have pointed out that thrombin treatment seems to negatively affect the yield of EVs isolation procedures since thrombin-induced clotting seems to entrap EVs leading to a reduction in the number of isolated EVs^15^. This reduction in the yield is consistent with our results, as thrombin treatment could reduce the number of recovered EVs, and therefore the number of miRNAs detected. On the other hand, NOR is a precipitation-based method followed by a purification step in a column chromatography. Our results indicate that NOR Kit on its complete procedure (exosome isolation plus RNA extraction) is well optimized. However, if NOR exosome isolation is combined with the phenol-based kit miRNeasy, this combination gives us less yield.

Regarding the RNA isolation kit, we observed that miRNeasy kit showed higer number of miRNAs. Similar results were found by Prendergast et al*.*^16^ in serum samples. Phenol-based methods such as miRNeasy kit usually performed well but seemed to produce some bias^17^, as structured smRNAs with low GC content are recovered inefficiently when using low RNA quantities.

Reports on smRNAs in EVs have been usually limited to miRNAs, but there is a variety of smRNAs, such as piRNAs, snoRNAs, snRNAs, and rRNAs, among others. PiRNAs were the most abundant after miRNAs. These molecules are a bit longer than miRNAs (24–30 nucleotides), and they are mainly expressed in germline cells but also in endothelial cells where their function is still unknown. PiRNAs are involved in DNA methylation and also mediate epigenetic changes; however, their primary function is suppressing transposable and other repetitive elements by binding to the PIWI subfamily of Argonaute proteins, keeping the genome integrity^18^. These smRNAs have been very little studied, but a putative implication in antimicrobial immunity has been explored in different organisms^19,20^. In this line, piwi proteins have recently been detected as inhibitors of HIV replication and other mobile genetic elements in activated T cells^21^, but additional studies need to be carried out to fully understand the piRNAs role in HIV and/or HCV infection.

Regarding the characterization of plasma EVs from HIV/HCV patients, we also analyzed the surface epitope markers of the plasma EVs, and the pathways regulated by their miRNA cargo. HIV-infected individuals have previously shown abundant plasma exosomes, enriched with CD9, CD63, and HSP70^22^, but there is no information about coinfection with HCV. On the one hand, we identified higher expression of exosome surface markers such as CD81, and molecules related to HIV and HCV infection, such as HLA-DR/DP/DQ and CD8. This is consistent with the higher immune activation of HIV/HCV coinfected patients. The MHC class II human leukocyte antigen isotypes (HLA-DR/DP/DQ) present antigens to immune cells, and they are widely present in exosomes, being also closely related to HIV and HCV infection. HLA-DP variants are related to the presentation of viral peptides, such as HIV envelope proteins^23^. HIV-induced immune activation leads to the expansion of CD8 T cells expressing HLA-DR, and the HLA-DR is incorporated into the envelope of HIV-1. The HLA-DR/DP/DQ molecules are also crucial determinants in the immune response to HCV infection, through the effective presentation of viral antigens to T cells. Thus, several studies have identified genomic variants at HLA region significantly associated with HCV persistence^24^. The CD81 is a tetraspanin involved in cell proliferation, especially enriched in the membrane of exosomes. This molecule is highly present in hepatocytes, being critical for HCV entry. CD81 is associated with the HCV envelope glycoprotein E2, which promotes the HCV transmission by exosomes^25^ within permissive cells, such as hepatocytes and peripheral blood mononuclear cells. CD81 is also involved in HIV assembly, co-localizing with the HIV-1 Gag protein in infected cells, exosomes, and virions^26^. Therefore, the surface markers identified in plasma EVs of HIV/HCV coinfected patients are associated with infectivity aspects of both viral infections.

Regarding the pathway enrichment analysis, fatty acid-related pathways were the main pathways targeted by the miRNA contained in EVs of HIV/HCV coinfected patients. Some of the miRNAs involved were hsa-miR-107, hsa-miR-103a-3p, hsa-miR-16-5p and hsa-miR-15a-5p, which targeted key fatty-acid genes such as *FASN, ACSL3, OXSM and ACSL4*. How these exosomal miRNAs are involved in modifying gene expression on target cells is still unknown, but they could be predictive biomarkers of liver disorders in HCV-related liver diseases. The interaction among HIV, HCV, and the antiretroviral treatment (ART) is associated with an increased risk of dyslipidemia, atherosclerosis, and cardiovascular diseases, among other metabolic abnormalities^27^. This deregulation may directly affect the innate antiviral response, liver disease progression, and the response to antiviral therapies. Additionally, HCV can hijacks and manipulates fatty acid flux to create specific lipid-enrichment microenvironments to promote its life cycle^28^, which will be reflected in the EVs content produced by the infected cells. We also identified the well-known miR-122 in plasma exosomes of HIV/HCV patients, which is a highly liver-specific miRNA implicated in fatty acids and cholesterol biosynthesis, among others. Additional significant pathways implicated in the fibrogenesis process were identified, such as are the glycosaminoglycan biosynthesis and the ECM receptors interactions pathways. The correct degradation of ECM is essential for the maintenance of tissue homeostasis, where the imbalance between the tissue inhibitors of metalloproteinases and matrix metalloproteinases causes liver damage and the progression or regression of fibrosis^29^. These molecules will, therefore, activate hepatic stellate cells (HSCs), which are the central mediator of fibrotic processes that will produce and accumulate extracellular matrix. This could explain, in part, the faster progression to fibrosis of HIV/HCV co-infected patients, since HIV creates a favorable milieu for the profibrogenic activation of HSCs^29^.

This is the first report exploring the exosome surface markers and the smRNA profile of HIV/HCV coinfected plasma-derived EVs. Currently, research on both viral infections with exosome interaction is preliminarily, and their mechanism of interaction needs further investigation.

## Conclusions

PEG precipitation-based protocols combined with a phenol-based RNA isolation kit is a suitable method for extracellular vesicles smRNA profiling of clinical samples. The RNA isolation kit also significantly impacts on the detection of miRNAs and other smRNAs, where the miRNEasy kit was appropriated in terms of the number of identified molecules. The plasma EVs of HIV/HCV patients are enriched with HLA-DR/DP/DQ, CD81 and CD8 surface molecules, and their miRNA cargo mainly modulates fatty-acid metabolism genes, among others.

## Material and methods

Twelve different experimental procedures were carried out for the purification of exosomes and other extracellular vesicles, and the isolation of the RNA for transcriptome analysis of smRNAs. Extended information on experimental design is included in Supplementary Material and Methods.

### Patients characteristics

Samples were recruited under the COVIHEP group as previously described^30^. Samples included in the present study were obtained from Hospital Universitario La Paz (Madrid) and processed at National Center for Microbiology, Institute of Health Carlos III, Madrid (Spain). The study protocol conformed to the ethical guidelines of the 1975 Declaration of Helsinki as reflected in a priori approval by the Institute of Health Carlos III review committee (CEI PI 67_2015-V4), and written informed consent was obtained from all patients involved.

Four HIV/HCV coinfected patients of Caucasian origin were recruited. All of them were receiving suppressive ART and showing CD4 + T-cells counts ≥ 500 cel/mm^3^ for at least one year before sample collection. Besides, patients had an active HCV-chronic infection, and they were naïve to any HCV treatment (positive PCR and positive HCV antibodies).

### Biological material

Whole peripheral blood was isolated and processed within the first 4 h after extraction. Density gradient centrifugation with Ficoll plaque was performed in order to separate peripheral blood mononuclear cells. Plasma fraction was pre-clarified at 15.000 rpm 15 min at 4 °C, and pooled and stored at – 80 °C until use.

### Bead-based multiplex exosome flow cytometry assay

Pooled plasma (clarified at 10.000 rpm 20 min at 4 °C and filtered with a 0.22 µm filter) was analyzed by bead-based multiplex EV analysis by flow cytometry (MACSPlex Exosome Kit, human, Miltenyi Biotec), following manufacturer instructions. This kit allows detection of 37 exosomal surface epitopes plus two isotype controls, each one can be distinguished by different fluorescence intensities. Briefly, samples were incubated with the antibody-coated MACSPlex Exosome Capture Beads, and they were labeled with the MACSPlex Exosome Detection Reagents (APC-conjugated anti-CD9, anti-CD63, and anti-CD81 detection antibody). Sandwich complexes were analyzed based on its fluorescence characteristics in a MACSQuant Analyzer 10 flow cytometer (Miltenyi Biotec), and the acquired data were analyzed with the Express Mode of the MACSQuantify Software v.2.11 (https://www.miltenyibiotec.com/US-en/products/macs-flow-cytometry/software/macsquantify/macsquantifytm-software.html#130-094-556).

Median fluorescence signal intensity (MFI) for all 39 capture bead subsets were background corrected by subtracting respective MFI values from the negative control. The normalization factor was performed by calculating the MFI of CD9, CD63, and CD81 markers for each sample.

### Exosome isolation protocols

In total, six different protocols were analyzed for its performance in miRNA and others smRNA recovery, from 1 ml of a pooled pre-clarified plasma previously frozen at – 80 °C.

#### Ultracentrifugation (UCF)

Plasma was centrifuged at higher speed (12,000×*g*), in order to remove macrovesicles and apoptotic bodies. Afterward, the plasma was ultracentifuged twice at 43,000 rpm using a Beckman Optima L-90 K Ultracentrifuge (SW55Ti rotor). The pellet of purified exosomes was resuspended in 100 μl of cold phosphate-buffered saline (PBS). This protocol was modified based on the contributions of Rekker et al.^31^ and Rani et al.^32^.

#### Total exosome isolation (TEI)

The total exosome isolation kit (from plasma) (ThermoFisher Scientific) was used according to the manufacturer´s instructions, with modifications recommended by Lane et al.^33^ and Van Deun et al.^34^***. ***Proteinase K treatment was performed to maximize the purity of the exosomes to eliminate plasma proteins and dissociating the extracellular ribonucleoprotein complexes.

#### Mircury (MIR)

The Mircury exosome isolation kit—Serum and Plasma, (Exiqon) was used according to the manufacturer´s instructions. This is a precipitation-based method which includes pretreatment of the plasma with thrombin to remove possible fibrin residues.

#### Norgen (NOR)

We used the commercial kit Norgen´s Plasma/Exosome Serum and Free-Circulating RNA Isolation Mini Kit (Norgen, Biotek corp.), to isolate exosomes by a precipitation-based method followed by a purification step in column chromatography. The kit was used according to the manufacturer's instructions.

#### Two custom polyethylene glycol (PEG) based protocols

Two custom protocols were used with different molecular weights of PEG: 6KDa (PEG6) and 10KDa (PEG10). PEG6 was performed as described by Andreu et al*.*^14^, PEG of 6,000 Da was previously prepared at 50% in 375 mM NaCl^14^. In the case of the PEG10, PEG 10,000 Da at 50% in deionized water was used^35^.

### Protein quantification

Samples were lysed in 2% Sodium Dodecyl Sulfate buffer in PBS with protease inhibitors (Complete Mini, Roche, Indianapolis, IN, USA) and protein concentration was determined by the Bicinchoninic acid protein assay (Pierce BCA protein assay, Thermo Scientific, Rockford, IL, USA). Total protein concentrations were determined using a standard linear curve established with bovine serum albumin.

### RNA extraction

Total RNA including smRNAs were extracted from previously isolated EVs using two different kits:

1. miRNeasy Mini kit (Qiagen), which is a phenol-based procedure, following manufacturer´s instructions. DNAse treatment was performed.
2. Plasma/Serum Exosome and Free-Circulating RNA Isolation Mini Kit (Norgen), which works for isolating any size of RNA from extracellular vesicles such as miRNAs and free-circulating RNAs, were used following the manufacturer´s instructions.

The RNA concentration was measured by Nanodrop, and RNA size distribution was evaluated by the Bioanalyzer 2100 with Agilent RNA 6000 Pico kit (Agilent, catalog no. 5067-1513).

### Sequencing

SmRNA library synthesis and sequencing were performed at the Centre for Genomic Regulation (CRG) at Barcelona (Spain). SmRNA library was performed with a high sensitivity kit for miRNA analysis in exosomes, the TailorMix miRNA Sample Preparation Kit (SeqMatic, ref. TM302), according to the manufacturer's protocol. Sequencing was performed in an Illumina HiSeq2500, single read, 50nts.

### Bioinformatic analysis

The miRNA data analysis was performed as previously described^28^. SmRNA data (snRNA, snoRNA, rRNA, and piRNAs) was extracted with Oasis 2 software^36^ from trimmed sequences.

### MiRNA quantification by qRT-PCR

MiRNAs were quantified in exosomes samples extracted by both the Qiagen and Norgen kits of exosomes isolated by all the different protocols as previously described^28^. Sequences of the miRNA nucleotides were extracted from the miRBase Release 21 (www.mirbase.org)^37^ (Supplementary Table S4).

### Statistical analysis

The statistical analyses were carried out with the R statistical package version v3.4.1 (R Foundation for Statistical Computing, Vienna, Austria).

The R-package “edgeR” (v 3.18.1)^38^ was used for the smRNA differential expression analysis. Trimmed mean of M-values normalization method was used, and a negative binomial generalized log-linear model to read counts for each gene. We used a GLM approach for multiple groups for determining differential expression among exosome protocols (two replicates per protocol), and among RNA isolation kits (six replicates per kit). It was performed with *glmQLFit()* and *glmQLFTest()* functions. Significantly differentially expressed (SDE) miRNAs were identified by a statistically significant p value < 0.05 adjusted by FDR using Benjamin-Hochberg correction, irrespective of the FC. We filtered out those miRNAs and smRNAs with less than the threshold of 12 counts among all samples.

We also performed a supervised multivariate analysis (multiple dependent variables) with a PLS-DA to create a regression model that classifies the variables according to their ability to classify each sample in the correct group. The optimal number of components for the model was determined with the leave-one-out cross-validation method using R2 and Q2 values as performance measures. All variables were previously normalized, log-transformed (generalized logarithm transformation), and auto-scaled (mean-centered and divided by the standard deviation of each variable). The PLS-DA also provides the VIP, which estimates the importance of each variable in the projection used in a PLS model. The VIP is used for ranking variables, where a VIP score greater than 1 is considered to enable discrimination between 2 groups.

A Venn diagram was performed to determine the overlapping SDE miRNAs between comparisons with InteractiVenn^39^.

### miRNA-based target prediction and pathway enrichment analysis of the target genes

The web-based computational tool DIANA-miRPath v3.0^40^ was used for the in silico target identification of the SDE miRNAs. This tool also performs a pathway union analysis of miRNAs targets, which is performed with the Kyoto Encyclopedia of Genes and Genomes (KEGG) pathways^41^.

## Supplementary information

Supplementary information 1

## Abbreviations

HCV: Hepatitis C virus
HIV: Human immunodeficiency virus
smRNA: Small RNA
EVs: Extracellular vesicles
ncRNAs: Non-coding RNAs
miRNAs: MicroRNAs
piRNAs: Piwi-interacting RNAs
snoRNAs: Small nucleolar RNAs
snRNAs: Small nuclear RNAs
rRNAs: Ribosomal RNAs
lncRNAs: Long non-coding RNAs
UCF: Ultracentrifugation
PEG: Polyethylene glycol
PEG10: Polyethylene glycol of 10 KDa
PEG6: Polyethylene glycol of 6 KDa
TEI: Total exosome isolation kit (Thermo Fisher Scientific)
MIR: Mircury exosome isolation kit—Serum and Plasma (Exiqon)
NOR: Norgen’s plasma/exosome serum and free-circulating RNA isolation Mini Kit (Norgen, Biotek corp.)
FC: Fold change
FDR: False discovery rate
PLS-DA: Partial least squares discriminant analysis
VIP: Variable importance in projection
MFI: Median fluorescence signal intensity
