# Supplementary material for: Comparison of methods and characterization of small RNAs from plasma extracellular vesicles of HIV/HCV coinfected patients
Source: Sci Rep. 2020 Jul 7;10:11140. doi: 10.1038/s41598-020-67935-1 (PMC7341746; doi:10.1038/s41598-020-67935-1)
Supplement: Supplementary file 1 — Supplementary information 1 [file 41598_2020_67935_MOESM1_ESM.docx]

# **SUPPLEMENTARY MATERIAL AND METHODS**

**Title: Characterization of small RNAs from plasma extracellular vesicles of HIV/HCV coinfected patients: a protocol comparison.**

**Authors names**: Martínez-González E.^1^, Brochado-Kith O^1^, Gómez-Sanz A.^1^, Martín-Carbonero L. ^2^, Jimenez-Sousa MA^1^, Martínez-Román P^1^, Resino S^1^, Briz V. ^1^*, Fernández-Rodríguez A^1^*.

*Both authors contributed equally

**Current affiliations:**

1. Unit of Viral Infection and Immunity, National Center for Microbiology, Institute of Health Carlos III, Majadahonda, Madrid, Spain.
2. Hospital La Paz Institute for Health Research (IdiPAZ), Madrid, Spain

## **Patients characteristics**

*Exclusion criteria*: no advanced liver fibrosis (>F3), clinical evidence of hepatic decompensation, active drug or alcohol addiction, alcohol-induced liver injury, HBV active infection, opportunistic infections, and other concomitant diseases such as diabetes, nephropathies, autoimmune disease, hemochromatosis, cryoglobulinemia, primary biliary cirrhosis, Wilson´s disease, α1-antitrypsin deficiency, and neoplasia.

## **Exosome Isolation protocols (extended version)**

We evaluated traditional protocols such as ultracentrifugation and polyethylene glycol (PEG) based procedures, and three commercial exosome isolation kits. In total six different protocols were analysed for its performance in microRNAs (miRNAs) and others small RNAs (smRNAs) recovery, from 1ml of frozen at -80°C pre-clarified plasma.

### 1 Ultracentrifugation (UCF):

Plasma was centrifugated at higher speed (12000g), in order to remove macrovesicles and apoptotic bodies. Afterwards, plasma was ultracentrifugated twice at 43,000 rpm on a Beckman Optima L-90K Ultracentrifuge (SW55Ti rotor). The pellet of purified exosomes was resuspended in 100 μl of cold phosphate-buffered saline (PBS). This protocol was modified based on the contributions of Rekker *et al*. (15) and Rani *et al*. (16), time and rpm conversions were made according to Beckman Coulter equivalences*.*

### 2. Total exosome isolation (TEI):

The Total Exosome Isolation kit from plasma (ThermoFisher Scientific) was used according to the manufacturer´s instructions, with modifications recommended by Lane *et al*. (17) and Van Deun *et al*. (18) ***.*** Proteinase K was added to the clarified plasma by incubating it at 37°C to eliminate plasma proteins, to maximize the purity of the exosomes and to dissociate the extracellular RNAs of protein complexes. Finally, exosome precipitation reagent) was added to precipitate the exosomes and incubated at 4°C overnight. Exosomes were resuspended in 100 μl of PBS.

### 3. Mircury (MIR):

The Mircury exosome isolation kit - Serum and Plasma, (Exiqon) is a precipitation-based method which include a pretreatment of the plasma with thrombin to remove possible fibrin residues. We followed manufacturer´s instruction, and exosomes were resuspended in 300 μl of Resuspension buffer under agitation at 37°C.

### 4. Norgen (NOR):

We used the commercial kit Norgen´s Plasma / Exosome Serum and Free-Circulating RNA isolation Mini Kit (Norgen, Biotek corp.), to isolate exosomes by a precipitation-based method followed by a purification step in a column chromatography. The kit was used according to the manufacturer's instructions.

### 5. Two custom polyethylene glycol (PEG) based protocols:

Two custom protocols were used with different molecular weights of PEG: 6KDa (PEG6) and 10 Kda (PEG10). The PEG6 was performed as described by Andreu *et al.* (19). Briefly; 1 ml of plasma was mixed with 400 μl of 50% PEG 6000 Da previously prepared in 375 mM NaCl. It was incubated 30 minutes on ice, centrifuged at 1500g for 30 minutes at 4°C and then the supernatant was discarded to remove the PEG residues. Finally, the pellet was resuspended in 200 μl of PBS. In the case of the PEG10, 50% PEG 10000 Da was also used, but in deionized water (20). 250 μl of PEG was added to 1 ml of plasma, incubated 2h at 4°C and centrifuged at 3000g and at 4°C. Finally, the pellet was resuspended in 200 μl of PBS.

## **Protein quantification**

Samples were lysed in 2% Sodium Dodecyl Sulfate buffer in PBS with protease inhibitors (Complete Mini, Roche, Indianapolis, IN, USA) and protein concentration was determined by the Bicinchoninic acid (BCA) protein assay (Pierce BCA protein assay, Thermo Scientific, Rockford, IL, USA). Total protein concentrations were determined using a standard linear curve established with bovine serum albumin.

## **RNA extraction**

Total RNA including smRNAs were isolated from plasma and Pheripheral blood mononuclear cells using two different kits:

4.1. miRNeasy Mini kit (Qiagen), which is a phenol-based procedure, following manufacturer´s instructions. Previously isolated exosomes were lysed by the addition of Qiazol. Then, ethanol was added to the aqueous phase to precipitate the RNA, according to the concentrations indicated by the manufacturer. Samples were also processed by silica columns where DNAse treatment was performed.

4.2. Plasma/Serum Exosome and Free-Circulating RNA Isolation Mini Kit (Norgen) which works for isolating any size of RNA from extracellular vesicles such as miRNAs and free-circulating RNAs, were used following manufacturer´s instructions.

RNA concentration was measured by Nanodrop and RNA size distribution was evaluated by the Bioanalyzer 2100 with Agilent RNA 6000 Pico kit (Agilent, catalog no. 5067-1513).

## **Sequencing**

SmRNA library synthesis and sequencing were performed at the Centre for Genomic Regulation at Barcelona (Spain). SmRNA library was performed with a high sensitivity kit for miRNA analysis in exosomes, the TailorMix miRNA Sample Preparation Kit (SeqMatic, ref. TM302), according to the manufacturer's protocol. Briefly, 3’ adapters and subsequently 5’ adapters were ligated to RNA. cDNA was synthesized and further amplified by PCR using indexed adapters. Finally, libraries were size selected using 6% Novex® TBE Gels (ref. EC6265BOX, Life Technologies). Fragments with insert sizes of 18 to 36 bp were selected, and DNA was precipitated and eluted in 10 µl elution buffer. Final libraries were analyzed using Agilent DNA 1000 chip to estimate the quantity and check size distribution, and were then quantified by qPCR using the KAPA Library Quantification Kit (ref. KK4835, KapaBiosystems) prior to amplification with Illumina’s cBot. Sequencing was performed in an Illumina HiSeq2500, Single Read, 50nts.

## **Bioinformatic analysis**

The raw data were initially filtered out for reads with ambiguous base calls, which did not meet the Illumina chastity filter based on quality measures and reads were sorted on sample type based on matches to the multiplex tags. Quality control of the remaining sequences was performed by using FastQC (v0.11.3)(21). Adapter sequences, as well as low quality base calls (q < 20), were trimmed with cutadapt (v. 1.13). Adapter trimmed reads were processed with miRDeep2 (v. 0.0.7) (22), which identify known and novel miRNAs from the dataset. This software map processed reads to the human reference genome (GRCh38) (mapper.pl module), based on Bowtie1. Only the alignments with 0 mismatches in the seed region and that do not map to more than five different loci in the genome were retained. Quantification was performed with quantifier.pl module, which maps the reads to miRNA precursors (obtained from miRBase v20 which contains 1917 precursors and 2654 mature sequences (23)), and determines the expression of the corresponding miRNAs. Only miRNAs with a minimum of 10 counts among all the samples were retained.

We use Oasis 2 software (24) for the identification of other smRNA species such as small nuclear RNAs (snRNA), small nucleolar RNAs (snoRNA), ribosomal RNAs (rRNA) and piwi-interacting RMAs (piRNAs). Trimmed sequences were uploaded and counts were identified.

## **MiRNA quantification by qRT-PCR**

MiRNAs were quantified in exosomes samples extracted by both the Qiagen and Norgen kits of exosomes isolated by all the different protocols. Total RNA was reverse transcribed into complementary DNA with the qScript microRNA cDNA synthesis kit using following manufacturer instructions. Briefly, miRNAs are polyadenylated, and the poly (A) tailed miRNAs are converted into first-stranded cDNA with an oligo-dT adapter primer. Individual miRNAs were quantified in a SYBR green quantitative PCR reaction by PerfeCta SYBR Green SuperMix (2x) (Quantabio) with a forward primer (sequence of the selected mature miRNA) and the PerfeCta universal PCR primer (specific to the unique sequence of the oligo-dT adapter primer). Sequences of the miRNA nucleotides were extracted from the miRBase Release 21 ([www.mirbase.org](http://www.mirbase.org)) (23) (**supplementary table 4**). PCR efficiency of each miRNA amplicon was evaluated with a standard curve using 10-fold dilution series of total RNA from a donor buffy. Only miRNA amplicons with an efficiency higher than 1.9 were accepted. Real time reactions were performed on triplicate in the Roche LightCycler 480.

## **miRNA-based target prediction and pathway enrichment analysis of the target genes**

The web-based computational tool DIANA-miRPath v3.0 (26) was used for the *in silico* target identification of the Significantly differentially expressed miRNAs. Analysis was based on experimentally supported targets predicted in silico, annotated in DIANA-TarBase v.8.0 (27). This tool also performs a pathway union analysis of those miRNAs targets, which is performed for Kyoto Encyclopedia of Genes and Genomes (KEGG) pathways (28). In addition, analysis of putative gene targets predicted in silico was performed with the microT- coding sequence(CDS) algorithm (29), which look for miRNA Recognition elements located in both the 3´-UTR and CDS regions. Enrichment p-values (Fischer-s exact test with hypergeometric distribution) were corrected for false discovery rate, and were considered significant when adjusted p ≤ 0.05.

# **SUPLEMENTARY TABLES**

## **Supplementary Table 1:** Total RNA concentration

|  | **RNA extraction kit (ng)** | |
| --- | --- | --- |
| **Exosome Isolation Protocol** | **Qiagen** | **Norgen** |
| UCF | 3.6 | 6.3 |
| TEI | 8.7 | 0.9 |
| MIR | 4.2 | 1.5 |
| NOR | 3 | 7.2 |
| PEG6 | 5.4 | 2.7 |
| PEG10 | 8.4 | 0.6 |

## **Supplementary Table 2:** Total miRNAs and other smRNAs identified and total count recovery with each experimental procedure. The total RNA was isolated from exosomes isolated by different protocols (UCF, TEI, MIR, NOR, PEG6, PEG10) with two different isolation kits: Norgen (MIRn, NORn, PEG10n, PEG6n, TEIn, UCFn) and Qiagen (MIRq, NORq, PEG10q, PEG6q, TEIq, UCFq) kit.

|  | **MIRn** | **MIRq** | **NORn** | **NORq** | **PEG10n** | **PEG10q** | **PEG6n** | **PEG6q** | **TEIn** | **TEIq** | **UCFn** | **UCFq** |
| --- | --- | --- | --- | --- | --- | --- | --- | --- | --- | --- | --- | --- |
| **miRNAs** |  |  |  |  |  |  |  |  |  |  |  |  |
| *number* | 97 | 118 | 83 | 77 | 122 | 156 | 93 | 152 | 130 | 154 | 122 | 92 |
| *counts* | 14416 | 8547 | 12195 | 13158 | 8264 | 6452 | 10869 | 6622 | 7520 | 6536 | 8264 | 10989 |
| **smRNAs** |  |  |  |  |  |  |  |  |  |  |  |  |
| *number* | 710 | 618 | 420 | 483 | 402 | 730 | 458 | 621 | 426 | 745 | 438 | 587 |
| *counts* | 1412 | 1623 | 2392 | 2079 | 2500 | 1373 | 2193 | 1615 | 2358 | 1346 | 2294 | 1709 |

## **Supplementary Table 3:** differentially expressed miRNAs and other smRNAs by RNA isolation kit (miRNeasy of Qiagen vs. Norgen).

| **miRNAs** | **logFC** | **logCPM** | **F** | **PValue** | **FDR** |
| --- | --- | --- | --- | --- | --- |
| hsa-let-7b-5p | 2.02 | 16.40 | 16.25 | 5.99E-05 | 0.012 |
| **smRNAs** | **logFC** | **logCPM** | **F** | **PValue** | **FDR** |
| hsa_piR_018734 | 9.25 | 14.51 | 49.60 | 1.86E-06 | ≤0.001 |
| hsa_piR_001043 | 9.01 | 13.53 | 33.32 | 2.16E-05 | 0.002 |
| hsa_piR_008033 | 9.13 | 11.63 | 28.94 | 4.81E-05 | 0.002 |
| hsa_piR_020185 | 9.82 | 9.98 | 28.87 | 4.88E-05 | 0.002 |
| RNU4-21P | 8.32 | 11.44 | 26.18 | 8.32E-05 | 0.002 |
| hsa_piR_015249 | 9.04 | 10.97 | 22.95 | 1.65E-04 | 0.004 |
| RNA5SP467 | 8.54 | 12.40 | 22.61 | 1.79E-04 | 0.004 |
| hsa_piR_002969 | 11.51 | 10.13 | 27.50 | 1.96E-04 | 0.004 |
| hsa_piR_021626 | -10.13 | 10.85 | 20.84 | 2.68E-04 | 0.004 |
| RNA5SP194 | 8.17 | 11.78 | 20.70 | 2.77E-04 | 0.004 |
| hsa_piR_011975 | 7.63 | 11.18 | 19.27 | 3.91E-04 | 0.005 |
| hsa_piR_011950 | 7.85 | 10.68 | 18.75 | 4.44E-04 | 0.005 |
| hsa_piR_011122 | 9.89 | 12.68 | 18.39 | 4.86E-04 | 0.005 |
| hsa_piR_006032 | 7.75 | 10.44 | 18.11 | 5.22E-04 | 0.005 |
| hsa_piR_018624 | 3.78 | 17.23 | 17.57 | 5.99E-04 | 0.006 |
| RNA5SP402 | 7.82 | 11.27 | 16.69 | 7.56E-04 | 0.007 |
| RNA5SP79 | 7.43 | 10.34 | 15.80 | 9.62E-04 | 0.008 |
| RNU4-32P | 6.99 | 10.70 | 15.76 | 9.71E-04 | 0.008 |
| hsa_piR_000159 | 9.58 | 16.25 | 15.53 | 1.03E-03 | 0.008 |
| hsa_piR_017153 | 7.63 | 11.21 | 14.96 | 1.22E-03 | 0.009 |
| RNU6-1163P | 7.08 | 10.03 | 14.20 | 1.51E-03 | 0.010 |
| hsa_piR_020388 | 7.95 | 11.10 | 13.85 | 1.67E-03 | 0.010 |
| RNA5SP188 | 7.67 | 10.15 | 13.80 | 1.69E-03 | 0.010 |
| hsa_piR_007567 | 7.00 | 10.33 | 13.74 | 1.73E-03 | 0.010 |
| hsa_piR_014306 | -2.50 | 6.81 | 13.72 | 1.74E-03 | 0.010 |
| RNA5SP39_RNA5SP395 | 7.88 | 12.13 | 13.44 | 1.88E-03 | 0.011 |
| RNA5SP265 | 5.71 | 9.74 | 13.25 | 2.00E-03 | 0.011 |
| RNA5SP338_RNA5SP33 | 8.19 | 12.80 | 12.44 | 2.55E-03 | 0.013 |
| RNA5SP210 | 8.49 | 11.84 | 11.97 | 2.95E-03 | 0.015 |
| hsa_piR_001184 | 5.85 | 14.22 | 10.49 | 4.78E-03 | 0.023 |
| SCARNA10 | 5.63 | 9.30 | 10.40 | 4.92E-03 | 0.023 |
| snoMe28S-Am2634 | 5.37 | 9.02 | 10.31 | 5.07E-03 | 0.023 |
| hsa_piR_005165 | -5.43 | 10.04 | 9.68 | 6.29E-03 | 0.028 |
| hsa_piR_020485 | 3.66 | 14.53 | 9.58 | 6.50E-03 | 0.028 |
| RNU5E-1_RNU5E-10P | 6.23 | 9.26 | 9.23 | 7.36E-03 | 0.031 |
| RNU2-50P | 6.43 | 8.98 | 9.10 | 7.72E-03 | 0.032 |
| hsa_piR_021256 | 4.92 | 12.84 | 8.68 | 8.97E-03 | 0.036 |
| RNA5SP286 | 5.42 | 9.43 | 8.61 | 9.19E-03 | 0.036 |
| SNORA45A | -6.67 | 12.29 | 8.24 | 1.05E-02 | 0.040 |
| hsa_piR_004276 | 5.18 | 9.20 | 7.59 | 1.34E-02 | 0.049 |

## **Supplementary Table 4:** miRNAs sequences for qRT-PCR of miRNAs

| **miRNA id** | **MiRBase miRNA sequence** | **Primer sequence** |
| --- | --- | --- |
| Hsa-miR-451a | AAACCGUUACCAUUACUGAGUU | AAACCGTTACCATTACTGAGTT |
| Hsa- miR-103a-3p | AGCAGCAUUGUACAGGGCUAUGA | AGCAGCATTGTACAGGGCTATGA |
| Hsa-miR-92a-3p | UAUUGCACUUGUCCCGGCCUGU | TATTGCACTTGTCCCGGCCTGT |
| Hsa-miR-484 | UCAGGCUCAGUCCCCUCCCGAU | TCAGGCTCAGTCCCCTCCCGAT |
| Hsa-miR-16-5p | UAGCAGCACGUAAAUAUUGGCG | TAGCAGCACGTAAATATTGGCG |
| Hsa-miR-150-5p | UCUCCCAACCCUUGUACCAGUG | TCTCCCAACCCTTGTACCAGTG |
| Hsa-miR-181a-5p | AACAUUCAACGCUGUCGGUGAGU | AACATTCAACGCTGTCGGTGAGT |
| Hsa-miR-423-3p | AGCUCGGUCUGAGGCCCCUCAGU | AGCTCGGTCTGAGGCCCCTCAGT |

**SUPLEMENTARY FIGURES**

## **Suplementary Figure 1:**

Total miRNA and other smRNAs normalized counts and number of different molecules by experimental procedure. Columns in red correspond to data from miRNeasy, Qiagen (Q), and columns in blue correspond to Norgen RNA isolation kit (N).

## **Suplementary Figure 2:**

Star Venn diagrams show the overlap of the smRNAs differentially identified between protocols. Each diagram have five ovals which contain the number of smRNAs differentially identified between each protocol versus **A)** UCF, **B)** TEI, **C)** MIR, **C)** NOR, **D)** PEG6 and **E)** PEG10. Overlapping numbers correspond to common smRNAs between different comparisons. Ovals colours correspond to comparison with UCF (pink), TEI (blue), MIR (orange), NOR (grey), PEG6 (yellow) and PEG10 (green).

**
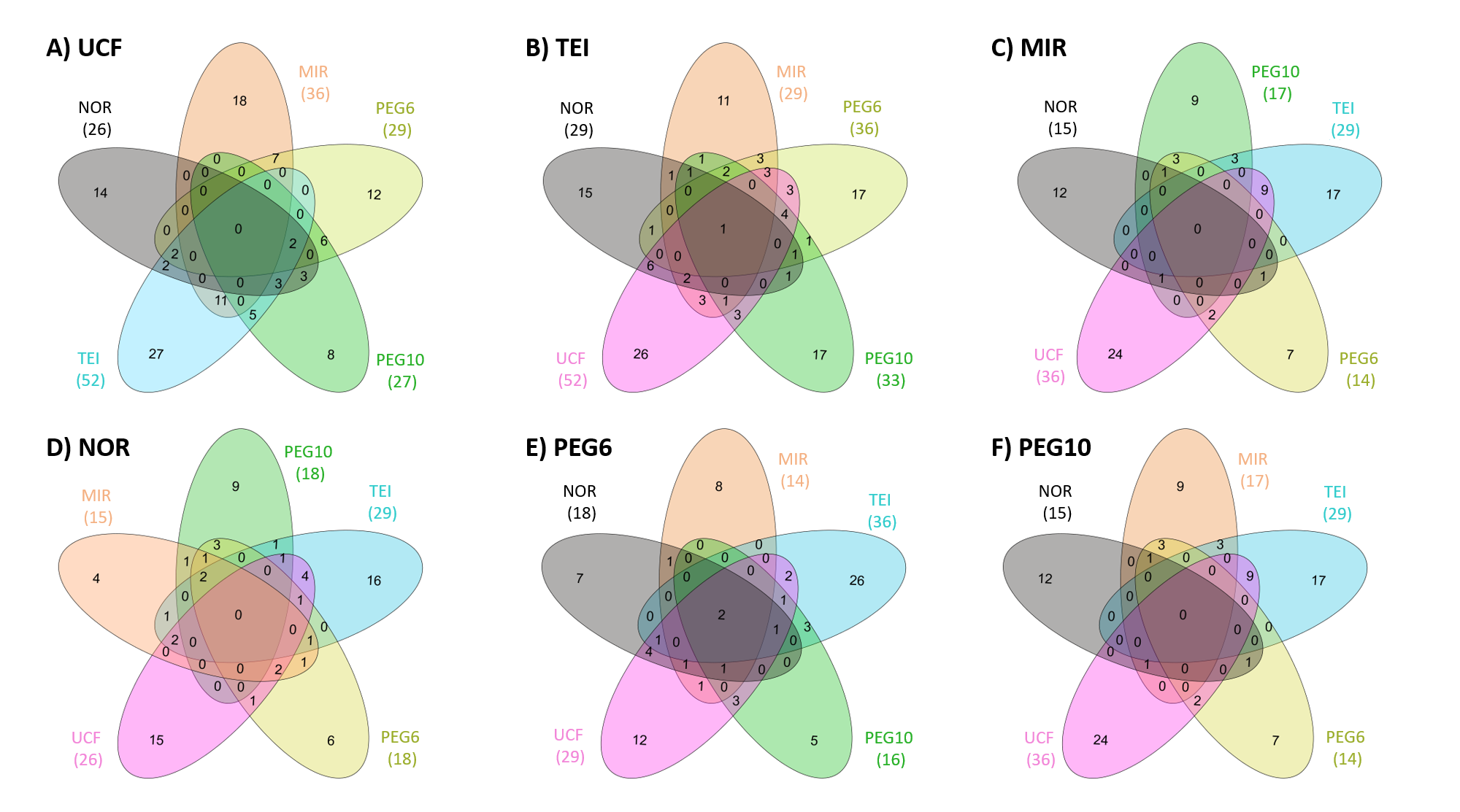
**

## **Supplementary Figure 3:**

Real time qPCR of selected miRNAs in all samples

**
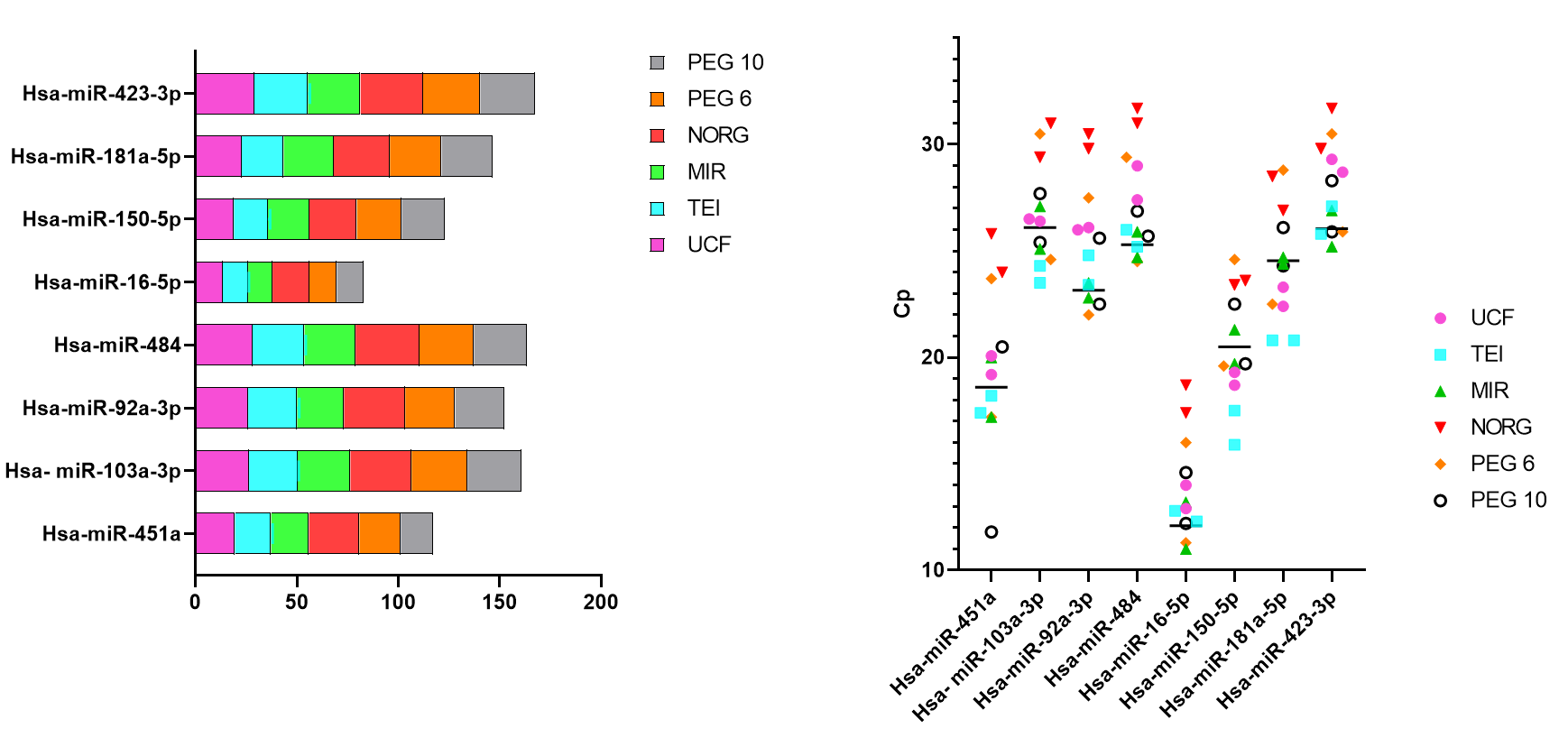
**
